# Short-term chemotherapy-related complications and undernutrition in children diagnosed with cancer at Korle Bu Teaching Hospital, Accra, Ghana: A prospective cohort study

**DOI:** 10.1371/journal.pone.0301208

**Published:** 2024-03-28

**Authors:** Nihad Salifu, Catherine I. Segbefia, Yakubu Alhassan, Lorna A. Renner, Edem M. A. Tette

**Affiliations:** 1 Department of Paediatrics, Greater Accra Regional Hospital, Accra, Ghana; 2 Department of Child Health, University of Ghana Medical School, Accra, Ghana; 3 Department of Biostatistics, School of Public Health, University of Ghana, Accra, Ghana; 4 Department of Community Health, University of Ghana Medical School, Accra, Ghana; Federal University of Agriculture Abeokuta, NIGERIA

## Abstract

Undernutrition in children with cancer is associated with complications during cancer therapy. The study objective was to determine the association between specific anthropometric parameters and short-term chemotherapy-related complications and mortality. This was a hospital-based, prospective cohort study of children, age ≤12 years, with a new cancer diagnosis at the Paediatric Oncology Unit, Korle Bu Teaching Hospital, Ghana. Socio-demographic information, cancer characteristics and anthropometric measurements were obtained at enrolment. Participants were followed up for twelve weeks from commencement of chemotherapy and selected treatment-related complications such as anaemia and thrombocytopenia requiring transfusions, prolonged neutropenia resulting in treatment delays, febrile neutropenia, mucositis and death were recorded. A total of 133 participants were recruited with a median age of 4.5 years. Eighty-one (60.9%) were diagnosed with solid tumours, 31 (23.3%) had leukaemias and 21 (15.8%) had lymphomas. Of the anthropometric parameters assessed, only arm anthropometry using upper arm muscle area (UAMA) and mid-upper arm circumference (MUAC) were associated with complications. Participants with wasting were more likely to develop anaemia and mucositis. However, the incidence of prolonged neutropenia was significantly higher among participants with average UAMA (p = 0.043) and low average UAMA (p = 0.049) compared to those with low UAMA. Risk of neutropenia was also significantly less among those with wasting by MUAC compared to those well-nourished (p = 0.045). Twenty-three participants (17.3%) died with a greater proportion (11/44; 25%) occurring in those who were wasted using MUAC. These findings underscore the need for nutritional surveillance at diagnosis and during chemotherapy, particularly where co-morbid disease is prevalent.

## Introduction

Five-year survival rates for childhood cancers in high-income countries (HIC) exceed 80% unlike low and middle-income countries (LMIC) where they are as low as 10% [1]. In 2018, the World Health Organization (WHO) launched the Global Initiative for Childhood Cancer to increase survival rates in LMIC to at least 60% by 2030. The initiative has the broad aims of prioritizing childhood cancers at policy level and building capacity to improve quality of care [2]. Recommended strategies to close the survival gap between HIC and LMIC include early diagnosis, reducing treatment abandonment and improving supportive care to decrease treatment-related toxicity [3, 4]. A prospective, observational cohort study across five Sub-Saharan African countries including Ghana, found that 15% of children died in the first 3 months of diagnosis, with 89% of those early deaths occurring from treatment-related causes [5].

The nutritional status of a child with cancer can be affected by the tumour or the treatment given. Nutritional status at diagnosis is an important modifiable factor for cancer treatment response [6]. In several studies, children with undernutrition have more severe bone marrow suppression than those who are well-nourished, resulting in higher rates of febrile neutropenia, prolonged neutropenia, severe anaemia and thrombocytopenia [6–9]. Undernutrition also leads to loss of mucosal barrier integrity and poor wound healing which increases the incidence, severity and duration of mucositis from chemotherapy [6]. Treatment delays can result from delayed bone marrow recovery, increased infection rates and reduced tolerance to treatment [6, 10]. Early recognition of malnutrition informs cancer treatment planning and enables appropriate adjustments to intensive chemotherapy regimens to minimize treatment-related toxicity and mortality [11].

The prevalence of undernutrition at diagnosis of cancer varies depending on several factors including the method of nutritional assessment. Anthropometric parameters are most commonly used in clinical settings and arm-based measurements such as the mid-upper arm circumference (MUAC) and upper arm muscle area (UAMA) are more sensitive than weight-based parameters in identifying undernutrition [11, 12]. The aim of this study was to determine the incidence of selected chemotherapy-related complications and early deaths in children diagnosed with cancer and the association with the anthropometric parameters used to assess the nutritional status at cancer diagnosis.

## Materials and methods

### Study design and setting

This was a hospital-based, prospective, observational cohort study at the Paediatric Oncology Unit (POU), Korle Bu Teaching Hospital (KBTH), Accra, Ghana. Participants were recruited at diagnosis of cancer and followed up for 12 weeks from the start of initial chemotherapy. Recruitment was from 4^th^ January, 2019 to 25^th^ December 2019, and the last follow up visit was on 18^th^ March 2020. KBTH is the largest hospital in Ghana and its POU is one of only two comprehensive childhood cancer treatment centres in the country. Children who require nutritional support are referred to the hospital dietetics department as there is no dietician assigned to the POU. At the time the study began, about 170 new cases were diagnosed each year and only children age ≤ 12 years were admitted to the POU due to a departmental policy.

Diagnosis of leukaemia was from bone marrow aspiration for morphology. Solid tumour diagnoses were from core needle or excision biopsies. Chemotherapy and supportive care costs, including nutritional rehabilitation, were out-of-pocket payments by families or philanthropists as childhood cancer services were not covered by the government-funded health insurance scheme.

### Sample size and sampling method

Sample size was calculated using Cochran’s formula, assuming a 5% (0.05) margin of error, 95% confidence level and undernutrition prevalence of 55% in children with cancer based on the study by Israels et al in Malawi [12, 13]. For feasibility, since the sample size (381) exceeded 5% of the average number of 170 newly diagnosed paediatric cancer cases presenting to the study site annually, it was corrected for a finite population resulting in a sample size of 118 [14, 15]. Allowing for 10% loss to follow-up, the final minimum sample size was ~132. Power analysis was performed using the exponential test for comparing two independent hazard rates approach in Stata IC version 16. Study participants were consecutively recruited until the sample size was achieved.

### Study population

All children aged ≤12 years with either of the following: (1.) a first, new diagnosis of cancer, or (2.) a second (different) cancer after completing treatment for a first cancer, or (3.) relapsed cancer after completion of cancer treatment and in confirmed remission for at least one year before relapse, were eligible for recruitment.

Children with chronic diseases such as tuberculosis or HIV infection and those who had started chemotherapy prior to recruitment were excluded.

### Data collection and procedure

The data record form included sociodemographic characteristics of participants including details of residential dwelling, household characteristics and access to a variety of consumer goods and services which were used to assess socioeconomic status [16]. Cancer characteristics (type, stage, and bone marrow involvement) were also documented. Due to limited capacity for molecular or cytogenetic studies, cancer risk group was assigned solely based on stage for lymphomas and solid tumours (low-risk: stages I & II and high-risk: stages III & IV). Stage IIB Hodgkin lymphoma was, however, categorized as high-risk [17]. Categorization of leukaemias as low- or high- risk was also rudimentary and based on previously published guidelines [18–20].

Baseline anthropometric measurements were performed by three trained research assistants and included weight, length or height, MUAC and triceps skinfold thickness (TSFT). Each measurement was obtained twice, and the mean value documented. Weight was measured to the nearest 0.1kg using a Seca 786® column dial scale (age ≥2 years) or a Seca 232® top loading infant weighing scale (age < 2 years). Length and height were measured to the nearest 0.1cm using a Hopkins measure mat class II® (serial number 680210) and the height gauge of class III Seca 786® column dial scale, respectively. The MUAC was measured with a non-stretchable three colour-coded Wintape® Shakir tape for participants aged 6 months to 5 years and the non-colour, non-stretchable MUAC tape for participants above age 5 years. The TSFT was measured with a pair of Harpenden skinfold callipers (John Bull British Indicators, Ltd). All measuring instruments were calibrated by the Ghana Standards Authority.

The WHO Anthro version 3.2.2 (for age < 5 years) and AnthroPlus version 1.0.4 (for age ≥ 5 years) software generated z-scores from weight, height or length and body mass index [21, 22]. The z-scores were used to classify stunting (height/length-for-age z score, HAZ), wasting (weight-for-height/length z score, WHZ), and thinness (BMI-for-age z score, BAZ) according to WHO 2007 growth standards and reference charts [23]. The MUAC was used to identify wasting and a participant was classified as malnourished if their age and sex-specific z score was less than the -2 z score. For participants age 6 months to 59 months, interpretation was based on the WHO arm anthropometry charts [23] while the MUAC reference curves by Mramba et al were used for those ≥ age 5 years [24]. For participants age ≥1 year, the UAMA was calculated from the MUAC and TSFT using the formula (MUAC– πTSFT)^2^/4π. UAMA percentile was obtained using the Frisancho percentile charts [25] and interpreted as follows: low/wasted: 0 to 5^th^ percentile, low average: 5.1 to 15^th^ percentile, average: 15.1 to 85^th^ percentile, above average: 85.1 to 95^th^ percentile, and high muscle: 95.1 to 100^th^ percentile [25]. The clinical team was informed of each participant’s nutritional status after assessment.

### Follow up

Participants were followed for 12 weeks from the start of chemotherapy during routine clinic visits and periods of acute illness. The following selected chemotherapy-related complications were assessed during follow-up: i) anaemia requiring red blood cell transfusion, ii) thrombocytopenia requiring platelet transfusion and/or resulting in delay of scheduled chemotherapy, iii) febrile neutropenia, iv) neutropenia resulting in delay of scheduled chemotherapy, and v) oral mucositis. All complications (except mucositis) were graded using the National Cancer Institute Common Terminology Criteria for Adverse Events, version 5.0 [26]. Oral mucositis was graded using the WHO criteria [27]. The intervention required for the complication and the length of chemotherapy interruption during the complication were documented. Mortalities during the study period were also documented.

### Statistical analysis

Data record forms were reviewed manually for accuracy and completeness. All data were entered and cleaned in Microsoft Excel 2016. Data were analyzed using Stata IC version 16 (Stata Corp, College Station, TX, USA). The socioeconomic status was generated into five wealth quintiles based on household expenditure and income, with the first and fifth quintiles being the lowest and highest levels, respectively [16]. Descriptive statistics (median, interquartile range, frequencies, and associated percentages) were used to summarize continuous and categorical variables. The Kaplan Meier hazard curve was used to describe the rate of development of treatment-related complications: (i) across the grades of UAMA at diagnosis, and (ii) based on MUAC classification (well-nourished vs. malnourished). The log-rank test was used to estimate equality of probability curves of the various treatment-related complications across UAMA and MUAC categories.

The Cox-proportional hazard model was used to assess the hazard rates of complications by nutritional status of participants at baseline. The sex, age group and cancer risk group were adjusted as confounding variables whilst nutritional status categorized by UAMA and MUAC were expressed as the exposure variables in the multivariable cox-proportional hazard model. The level of statistical significance was p<0.05.

### Ethical considerations

Ethical approval was granted by the Institutional Review Board (IRB) of KBTH (KBTH-IRB 000112/2018). Written, informed consent was obtained from all caregivers and assent from participants age ≥8 years.

## Results

**Fig 1:**
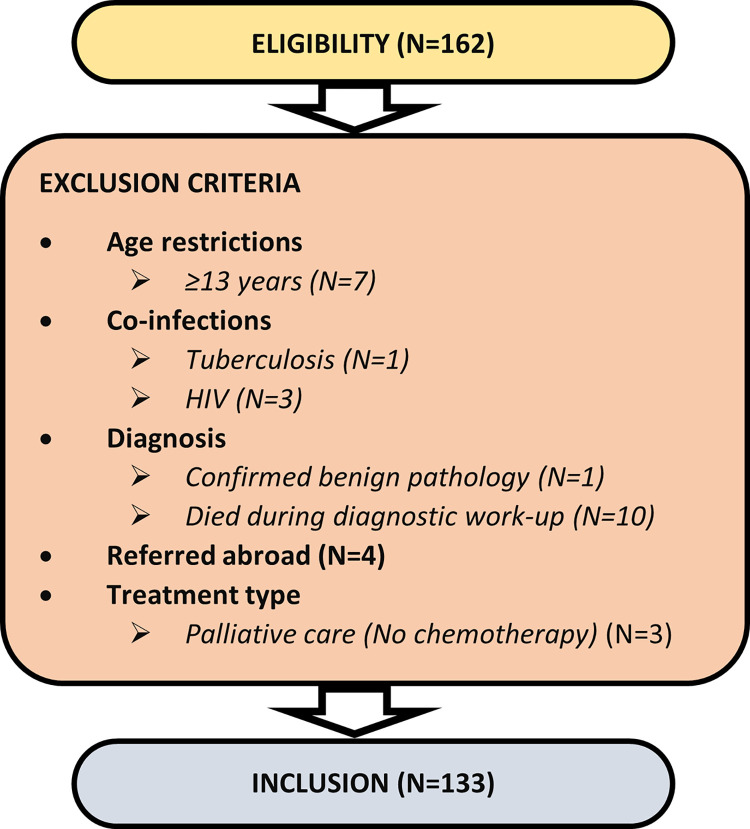
Study eligibility and exclusion criteria.

From January to December 2019, 162 children were referred to the POU with a suspected diagnosis of cancer. Of these, 133 met study eligibility criteria and were recruited and 99/133 (74.4%) completed the 12-week follow up period (Fig 1). All recruited participants had a first, new diagnosis of cancer. Seventy (52.6%) were <age 5 years and 86 (64.7%) were males. Eighty-one (60.9%) children had solid tumours, 31 (23.3%) had leukaemias and 21 (15.8%) had lymphomas.

Overall, 94 (70.7%) were categorized as having high risk disease. Of the 102 children diagnosed with solid tumours and lymphomas, 53 (52.0%) had initial bone marrow testing and 12 out of the 53 (22.6%) had confirmed bone marrow involvement. Forty-nine children did not have initial bone marrow testing done for various reasons such as the child being too ill for the procedure and a bone marrow test not required routinely for staging specific solid tumours e.g., osteosarcoma, hepatoblastoma etc.

Participants’ sociodemographic characteristics and nutritional categories at diagnosis by cancer group are shown in Table 1. Using weight-based parameters (WHZ and BAZ), over a fifth (29/133; 21.8%) of study participants were undernourished (wasted and/or thin, respectively). Arm anthropometric parameters (MUAC and UAMA) identified wasting in a higher proportion of participants (Table 1) and 22/133 (16.5%) were stunted.

**Table 1:** Sociodemographic characteristics and nutritional categories at diagnosis of cancer.

		Cancer group			
	Total	Leukaemia	Lymphoma	Solid tumour	Chi-square P-value
Characteristics	N = 133	N = 31	N = 21	N = 81	
	n/N (%)	n/N (%)	n/N (%)	n/N (%)	
**Age group of children**					0.003
<12 months	10/133 (7.5)	2/31 (6.5)	0/21 (0.0)	8/81 (9.9)	
12 to 59 months	60/133 (45.1)	15/31 (48.4)	3/21 (14.3)	42/81 (51.9)	
60 to 119 months	34/133 (25.6)	4/31 (12.9)	11/21 (52.4)	19/81 (23.5)	
120 to 155 months	29/133 (21.8)	10/31 (32.3)	7/21 (33.3)	12/81 (14.8)	
**Sex of child**					0.390
Male	86/133 (64.7)	23/31 (74.2)	14/21 (66.7)	49/81 (60.5)	
Female	47/133 (35.3)	8/31 (25.8)	7/21 (33.3)	32/81 (39.5)	
**Place of residence**					0.036
Greater Accra	58/133 (43.6)	19/31 (61.3)	5/21 (23.8)	34/81 (42.0)	
Outside Greater Accra	70/133 (52.6)	10/31 (32.3)	16/21 (76.2)	44/81 (54.3)	
Foreigner	5/133 (3.8)	2/31 (6.5)	0/21 (0.0)	3/81 (3.7)	
**Household wealth category**					0.280
Poorest	27/133 (20.3)	4/31 (12.9)	6/21 (28.6)	17/81 (21.0)	
Very poor	27/133 (20.3)	4/31 (12.9)	5/21 (23.8)	18/81 (22.2)	
Poor	26/133 (19.5)	9/31 (29.0)	6/21 (28.6)	11/81 (13.6)	
Less poor	27/133 (20.3)	6/31 (19.4)	2/21 (9.5)	19/81 (23.5)	
Least poor	26/133 (19.5)	8/31 (25.8)	2/21 (9.5)	16/81 (19.8)	
**Cancer group**					-
Leukaemia	31/133 (23.3)	31/31 (100.0)	-	-	
Lymphoma	21/133 (15.8)	-	21/21 (100.0)	-	
Solid tumour	81/133 (60.9)	-	-	81/81 (100.0)	
**Cancer risk group**					0.080
High risk	94/133 (70.7)	22/31 (71.0)	19/21 (90.5)	53/81 (65.4)	
Low risk	39/133 (29.3)	9/31 (29.0)	2/21 (9.5)	28/81 (34.6)	
**Bone marrow involvement**					<0.001
No	41/133 (30.8)	0/31 (0.0)	12/21 (57.1)	29/81 (35.8)	
Yes	43/133 (32.3)	31/31 (100.0)	0/21 (0.0)	12/81 (14.8)	
Not done	49/133 (36.8)	0/31 (0.0)	9/21 (42.9)	40/81 (49.4)	
**UAMA at diagnosis**					0.024
Low	50/133 (37.6)	11/31 (35.5)	15/21 (71.4)	24/81 (29.6)	
Low average	30/133 (22.6)	6/31 (19.4)	4/21 (19.0)	20/81 (24.7)	
Average	43/133 (32.3)	12/31 (38.7)	2/21 (9.5)	29/81 (35.8)	
N/A	10/133 (7.5)	2/31 (6.5)	0/21 (0.0)	8/81 (9.9)	
**HFA at diagnosis**					0.510
Not stunted	109/133 (82.0)	27/31 (87.1)	15/21 (71.4)	67/81 (82.7)	
Stunted	22/133 (16.5)	4/31 (12.9)	5/21 (23.8)	13/81 (16.0)	
N/A	2/133 (1.5)	0/31 (0.0)	1/21 (4.8)	1/81 (1.2)	
**WFH at diagnosis**					0.007
Not wasted	50/133 (37.6)	13/31 (41.9)	2/21 (9.5)	35/81 (43.2)	
Wasted	17/133 (12.8)	3/31 (9.7)	1/21 (4.8)	13/81 (16.0)	
N/A	66/133 (49.6)	15/31 (48.4)	18/21 (85.7)	33/81 (40.7)	
**BMI at diagnosis**					0.610
Not thin	101/133 (75.9)	26/31 (83.9)	14/21 (66.7)	61/81 (75.3)	
Thin	29/133 (21.8)	5/31 (16.1)	6/21 (28.6)	18/81 (22.2)	
N/A	3/133 (2.3)	0/31 (0.0)	1/21 (4.8)	2/81 (2.5)	
**MUAC at diagnosis**					0.008
Well-nourished	87/133 (65.4)	23/31 (74.2)	7/21 (33.3)	57/81 (70.4)	
Malnourished	44/133 (33.1)	8/31 (25.8)	14/21 (66.7)	22/81 (27.2)	
Not applicable	2/133 (1.5)	0/31 (0.0)	0/21 (0.0)	2/81 (2.5)	

UAMA, upper arm muscle area; HFA, height for age; WFH, weight for height; BMI, body mass index; MUAC, mid upper arm circumference.

### Nutritional status and risk factors for chemotherapy-related complications

All 133 children enrolled into the study were included in the analysis. Those who did not complete follow-up due to abandonment (n = 11, 8.3%) or death (n = 23, 17.3%) had their data censored and added to final analysis. The average number of follow up weeks for participants who abandoned treatment was 3.5±1.8 weeks. Four complications occurred in two participants who abandoned treatment: two episodes of anaemia and one episode each of neutropenia and thrombocytopenia. Overall, 90 participants (67.7%) had complications, while 43 (32.3%) had none. Of those who had complications, 35/90 (38.9%) had one complication, 30/90 (33.3%) had two complications and 25/90 (27.8%) had three or more complications. The commonest complication was anaemia (36.8%) while oral mucositis (7.5%) was the least common (Table 2). Four out of the ten participants (40%) who developed mucositis had febrile neutropenia concurrently. A total of 30 episodes of prolonged neutropenia occurred in 23 participants while 33 episodes of febrile neutropenia occurred in 26 participants. The average time of treatment delay for prolonged neutropenia and febrile neutropenia were 10.6±5.6 days and 5.0±2.0 days, respectively. Overall incidence of complications among participants with solid tumours was 60% less than those with leukaemias (IRR: 0.40, 95% CI: 0.23–0.70, p = 0.001). Anaemia, febrile neutropenia, and prolonged neutropenia were significantly less frequent among participants with solid tumours. (Table 2).

**Table 2:** Incidence of complications within 12 weeks of cancer treatment among participants by cancer groups.

			Incidence rate per 100 person weeks	Incidence rate ratio	
Complications & cancer groups	n/N (%)	Total person times (weeks)	IR [95% CI]	IRR [95% CI]	P-value
**Any complications**					
All cancers	90/133 (67.7)	717.1	12.5 [10.2, 15.4]		
Leukaemia	27/31 (87.1)	111.0	24.3 [16.7, 35.5]	1.00 [reference]	
Lymphoma	14/21 (66.7)	100.0	14.0 [8.3, 23.6]	0.58 [0.26, 1.30]	0.182
Solid tumour	49/81 (60.5)	506.1	9.7 [7.3, 12.8]	0.40 [0.23, 0.70]	0.001
**Anaemia**					
All cancers	49/133 (36.8)	993.6	4.9 [3.7, 6.5]		
Leukaemia	20/31 (64.5)	188.0	10.6 [6.9, 16.5]	1.00 [reference]	
Lymphoma	7/21 (33.3)	131.1	5.3 [2.5, 11.2]	0.50 [0.20, 1.27]	0.147
Solid tumour	22/81 (27.2)	674.4	3.3 [2.1, 5.0]	0.31 [0.16, 0.58]	<0.001
**Febrile Neutropenia**					
All cancers	39/133 (29.3)	1041.7	3.7 [2.7, 5.1]		
Leukaemia	18/31 (58.1)	193.4	9.3 [5.9, 14.8]	1.00 [reference]	
Lymphoma	6/21 (28.6)	151.7	4.0 [1.8, 8.8]	0.42 [0.16, 1.11]	0.081
Solid tumour	15/81 (18.5)	696.6	2.2 [1.3, 3.6]	0.23 [0.11, 0.49]	<0.001
**Neutropenia**					
All cancers	33/133 (24.8)	1093.9	3.0 [2.1, 4.2]		
Leukaemia	14/31 (45.2)	212.7	6.6 [3.9, 11.1]	1.00 [reference]	
Lymphoma	5/21 (23.8)	147.4	3.4 [1.4, 8.1]	0.52 [0.18, 1.46]	0.213
Solid tumour	14/81 (17.3)	733.7	1.9 [1.1, 3.2]	0.29 [0.14, 0.62]	0.001
**Thrombocytopenia**					
All cancers	29/133 (21.8)	1193.9	2.4 [1.7, 3.5]		
Leukaemia	9/31 (29.0)	268.0	3.4 [1.7, 6.5]	1.00 [reference]	
Lymphoma	3/21 (14.3)	169.4	1.8 [0.6, 5.5]	0.53 [0.15, 1.85]	0.318
Solid tumour	17/81 (21.0)	756.4	2.2 [1.4, 3.6]	0.67 [0.31, 1.43]	0.301
**Mucositis**					
All cancers	10/133 (7.5)	1253.3	0.8 [0.4, 1.5]		
Leukaemia	2/31 (6.5)	303.3	0.7 [0.2, 2.6]	1.00 [reference]	
Lymphoma	3/21 (14.3)	166.9	1.8 [0.6, 5.6]	2.73 [0.45, 16.44]	0.274
Solid tumour	5/81 (6.2)	783.1	0.6 [0.3, 1.5]	0.97 [0.18, 5.11]	0.970
**Death**					
All cancers	23/133 (17.3)	1315.1	1.7 [1.2, 2.6]		
Leukaemia	6/31 (19.4)	312.0	1.9 [0.9, 4.3]	1.00 [reference]	
Lymphoma	7/21 (33.3)	183.7	3.8 [1.8, 8.0]	1.98 [0.64, 6.13]	0.236
Solid tumour	10/81 (12.3)	819.4	1.2 [0.7, 2.3]	0.63 [0.22, 1.83]	0.399

**Fig 2:**
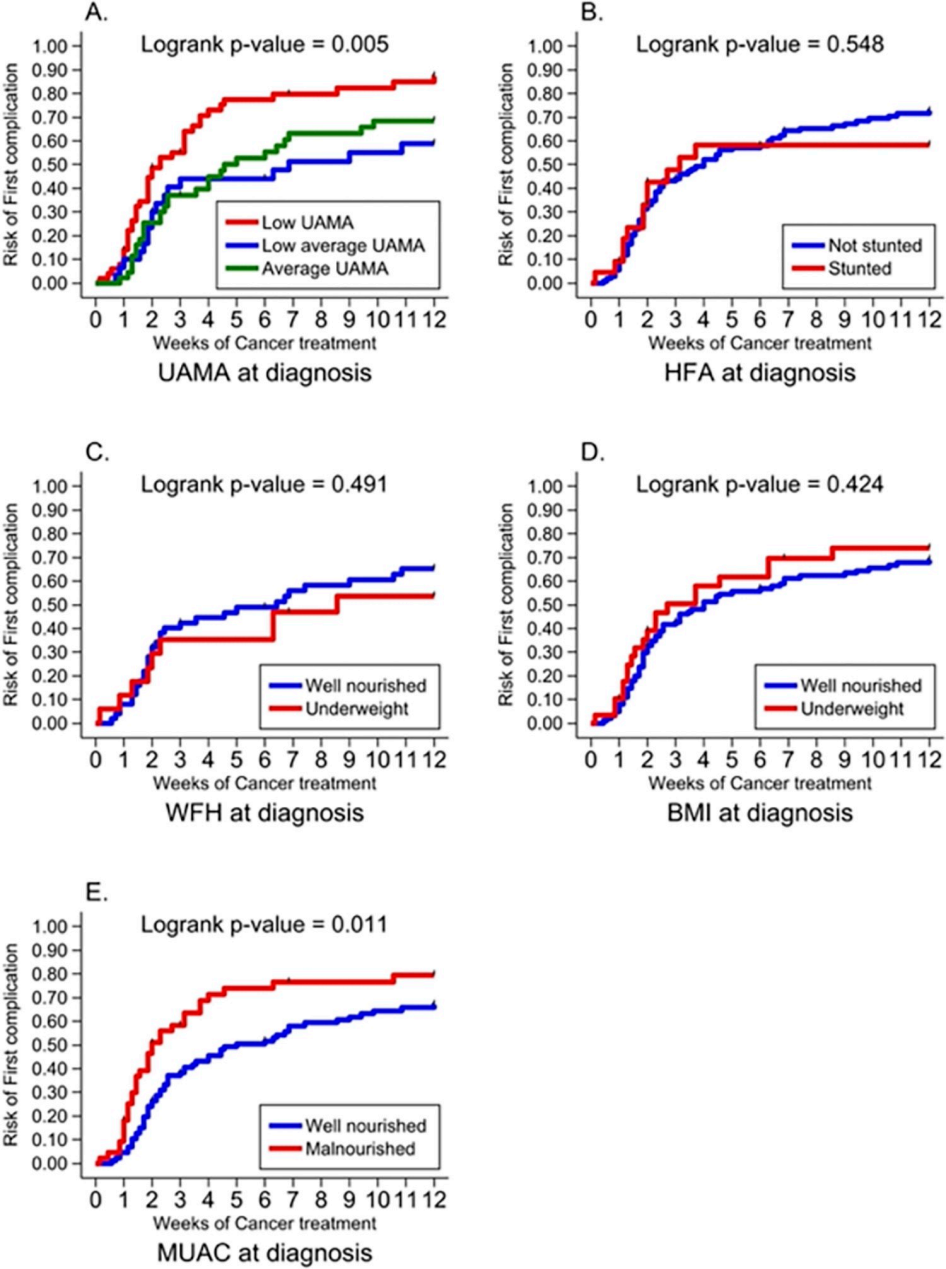
Risk of developing complications based on nutritional status at diagnosis using different anthropometric parameters.

**Fig 3:**
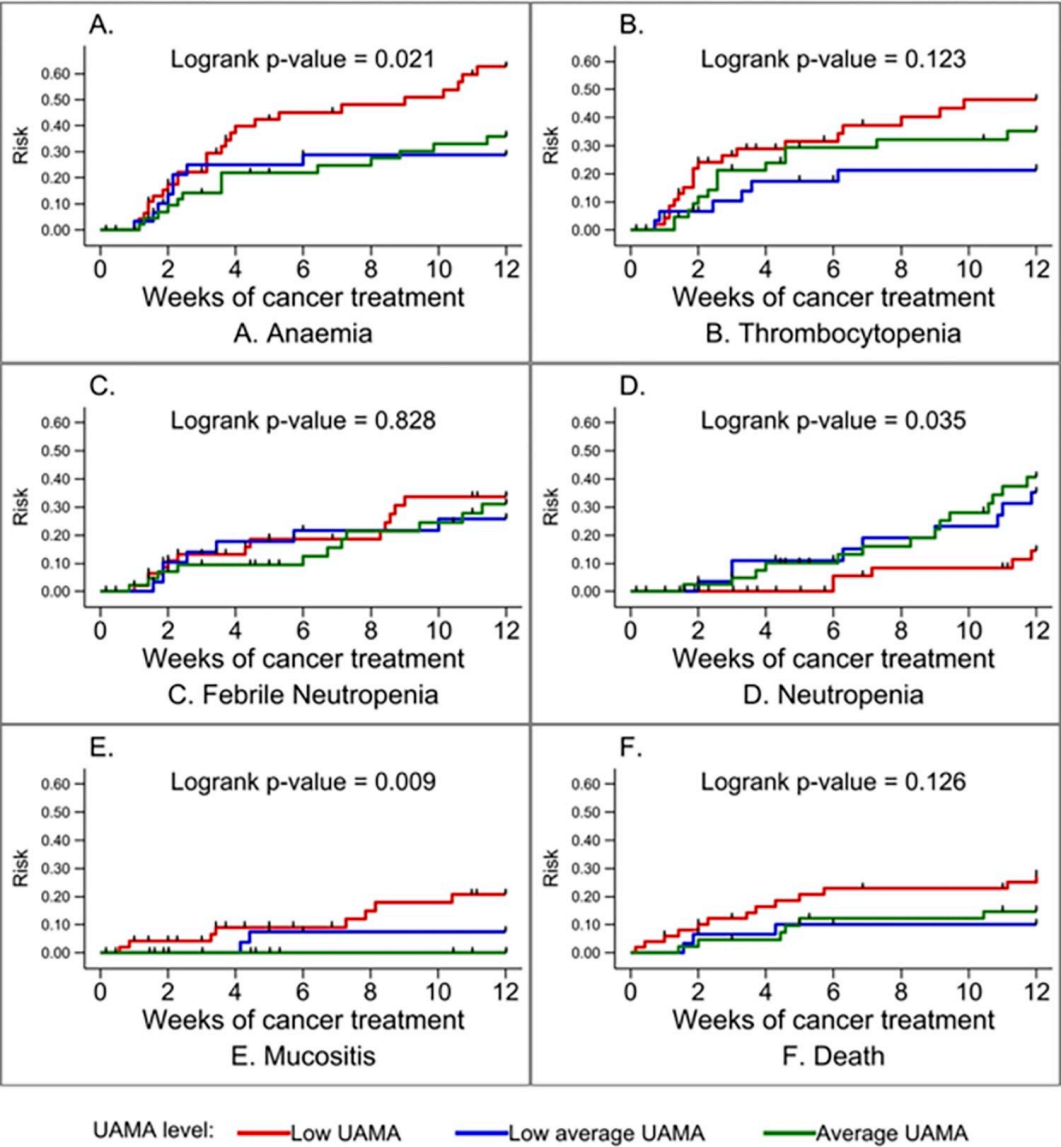
Kaplan Meier hazard curves for specific complications across the grades of upper arm muscle area (UAMA) at diagnosis.

**Fig 4:**
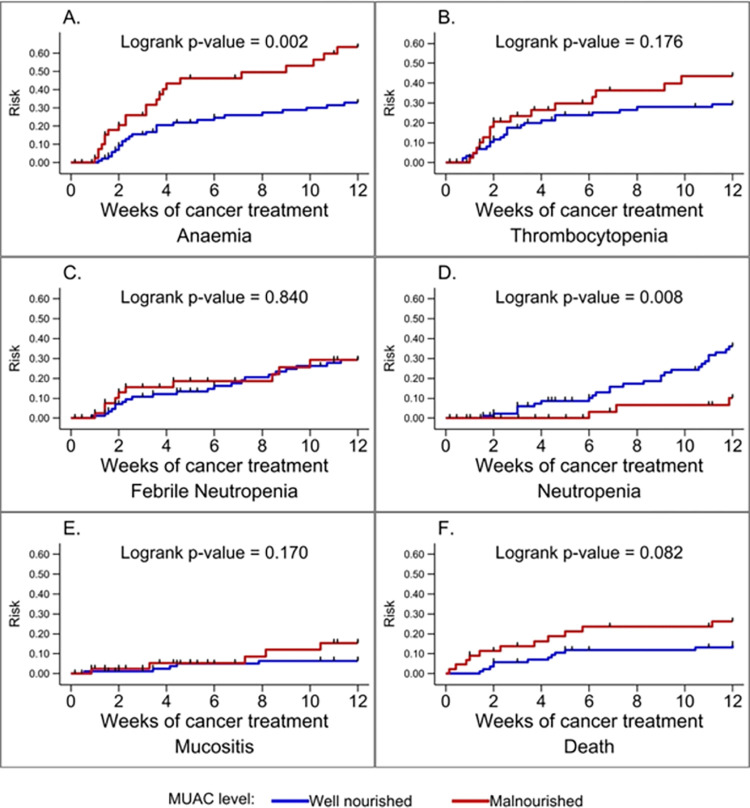
Kaplan Meier hazard curves for specific complications based on mid-upper arm circumference (MUAC) classification at diagnosis.

None of the weight-based anthropometric parameters (WHZ/BAZ) or HAZ were associated with development of complications (Fig 2). UAMA grade and MUAC were significantly associated with a higher overall incidence of complications. Participants with low UAMA had a higher risk of anaemia and mucositis (Fig 3). No participant from the average UAMA group developed mucositis. In contrast, those with average UAMA had the highest risk of neutropenia (Fig 3). Participants classified as malnourished by MUAC also had a significantly higher risk of anaemia. Conversely, those who were well-nourished had a higher risk of prolonged neutropenia (Fig 4). Nutritional status did not affect the risk of developing thrombocytopenia or febrile neutropenia.

In the adjusted model, neither UAMA grade nor MUAC showed a significant association with the risk of developing anaemia (Table 3). The risk of neutropenia however remained significantly higher among children with average UAMA (aHR: 2.76, 95% CI: 1.03–7.37, p = 0.043) and low average UAMA (aHR: 2.84, 95% CI: 1.00–8.01, p = 0.049) compared to those with low UAMA. The risk of neutropenia was also significantly less among those who were malnourished by MUAC compared to those well-nourished (aHR: 0.29, 95% CI: 0.09–0.97, p = 0.045).

**Table 3:** Cox-proportional hazard model of the association between nutritional status and selected complications among children with cancer during the first 12 weeks of treatment.

	Outcomes	Unadjusted Cox model		Adjusted Cox model	
Exposure	n/N (%)	uHR [95% CI]	P-value	aHR [95% CI]	P-value
**UAMA level**	**Any complication**				
Low	41/50 (82.0)	1.00 [reference]		1.00 [reference]	
Low average	17/30 (56.7)	0.57 [0.32, 0.99]	0.048	0.80 [0.40, 1.59]	0.526
Average	28/43 (65.1)	0.68 [0.42, 1.09]	0.111	0.94 [0.49, 1.80]	0.851
**MUAC**					
Well-nourished	56/87 (64.4)	1.00 [reference]		1.00 [reference]	
Malnourished	33/44 (75.0)	1.73 [1.12, 2.66]	0.013	1.51 [0.79, 2.88]	0.209
**UAMA level**	**Anaemia**				
Low	25/50 (50.0)	1.00 [reference]		1.00 [reference]	
Low average	8/30 (26.7)	2.24 [1.19, 4.20]	0.012	1.27 [0.47, 3.45]	0.633
Average	14/43 (32.6)	0.93 [0.40, 2.18]	0.870	1.02 [0.43, 2.40]	0.966
**MUAC**				-	-
Well-nourished	26/87 (29.9)	1.00 [reference]		-	-
Malnourished	22/44 (50.0)	2.34 [1.35, 4.08]	0.003	1.75 [0.69, 4.44]	0.238
**UAMA level**	**Thrombocytopenia**				
Low	5/50 (10.0)	1.00 [reference]		1.00 [reference]	
Low average	9/30 (30.0)	1.82 [0.91, 3.64]	0.089	1.98 [0.68, 5.78]	0.209
Average	14/43 (32.6)	0.74 [0.28, 1.91]	0.528	0.83 [0.31, 2.20]	0.706
**MUAC**					
Well-nourished	26/87 (29.9)	1.00 [reference]		1.00 [reference]	
Malnourished	3/44 (6.8)	1.59 [0.83, 3.05]	0.155	0.85 [0.31, 2.30]	0.742
**UAMA level**	**Febrile neutropenia**				
Low	19/50 (38.0)	1.00 [reference]		1.00 [reference]	
Low average	6/30 (20.0)	0.83 [0.34, 2.04]	0.690	1.02 [0.34, 3.01]	0.952
Average	14/43 (32.6)	0.92 [0.42, 2.01]	0.836	1.06 [0.38, 2.95]	0.912
**MUAC**					
Well-nourished	26/87 (29.9)	1.00 [reference]		1.00 [reference]	
Malnourished	3/44 (6.8)	1.07 [0.51, 2.25]	0.862	0.99 [0.35, 2.79]	0.912
**UAMA level**	**Neutropenia**				
Low	5/50 (10.0)	1.00 [reference]		1.00 [reference]	
Low average	9/30 (30.0)	3.08 [1.10, 8.65]	0.033	2.84 [1.00, 8.01]	0.049
Average	14/43 (32.6)	3.63 [1.39, 9.45]	0.008	2.76 [1.03, 7.37]	0.043
**MUAC**					
Well-nourished	26/87 (29.9)	1.00 [reference]		1.00 [reference]	
Malnourished	3/44 (6.8)	0.24 [0.07, 0.78]	0.017	0.29 [0.09, 0.97]	0.045
**UAMA level**	**Mucositis**				
Low	8/50 (16.0)	1.00 [reference]		1.00 [reference]	
Low average	2/30 (6.7)	0.18 [0.04, 0.83] ^+^	0.028	0.19 [0.03, 1.20] ^+^	0.077
Average	0/43 (0.0)	-	-	-	-
**MUAC**					
Well-nourished	5/87 (5.7)	1.00 [reference]		1.00 [reference]	
Malnourished	5/44 (11.4)	2.39 [0.69, 8.22]	0.169	0.60 [0.12, 2.82]	0.521
**UAMA level**	**Death**				
Low	5/50 (10.0)	1.00 [reference]		1.00 [reference]	
Low average	9/30 (30.0)	0.40 [0.11, 1.38]	0.146	0.58 [0.12, 2.84]	0.503
Average	14/43 (32.6)	0.56 [0.21, 1.45]	0.232	0.99 [0.23, 4.20]	0.984
**MUAC**				-	-
Well-nourished	12/87 (13.8)	1.00 [reference]		-	-
Malnourished	11/44 (25.0)	2.09 [0.92, 4.72]	0.078	0.78 [0.22, 2.87]	0.831

n/N (%): Number with outcome/Number in exposure group (Row percentage)

uHR, unadjusted hazard rate; aHR, adjusted hazard rate; CI, confidence interval; UAMA, upper arm muscle area; MUAC, mid upper arm circumference.

^+:^ Low and low average UAMA level were combined as a group to estimate hazard rate.

### Mortality among study participants and risk factors for mortality

There were 23 deaths during the follow up period comprising 17.3% of all 133 study participants and 23.2% of the 99 participants who completed 12 weeks of follow-up. The incidence of death during the study follow-up period was 1.7 (95% CI: 1.2–2.6) cases per 100 persons per week (Table 2). Of those who died, 10 (43.5%) had solid tumours, 7 (30.4%) had non-Hodgkin lymphoma, and 6 (26.1%) had acute leukaemia. All except one had cancers in the high-risk groups. Thirteen (57%) were wasted at diagnosis and 5 (21.7%) were stunted. Disease metastasis (central nervous system and/or lungs) was the cause of death in 10 (43.5%), while 4 (17.4%) died from intracranial haemorrhage, all 4 of whom had acute leukaemia. Eight of the 23 deaths (34.8%) were probable infections (no culture results were positive) and 1 (4.3%) was due to tumour lysis syndrome.

Nutritional status was not a risk factor for death even though participants with wasting by MUAC were more likely to die than those who were well-nourished (11/44; 25% vs. 12/87; 13.8%, respectively).

## Discussion

In this study, only nutritional status classified by arm anthropometry was associated with chemotherapy-related complications. Children with wasting by UAMA and those classified as malnourished using MUAC had an overall increased risk of developing a complication within the first 12 weeks of cancer treatment. These findings highlight the importance of incorporating arm anthropometry into standardized nutritional assessments of children at diagnosis of cancer instead of depending on weight-based measurements, especially in settings where high risk or advanced disease is common and weight-based measures are likely to be inaccurate. A hospital-based study in Kumasi, Ghana, and similar studies from other African countries have confirmed the increased sensitivity of arm anthropometric parameters such as MUAC, UAMA and TSFT (used alone or in combination), in identifying undernutrition, compared to weight-based measurements like WHZ [12, 28, 29]. In populations where there is a high baseline prevalence of stunting, WHZ will be interpreted as normal in children who are both underweight and stunted. Large tumour burden from advanced-stage disease which are common in LMICs and the presence of ascites and pedal oedema also falsely increase weight, confounding the interpretation of WHZ and BAZ.

The incidence of complications was significantly lower in those with solid tumours compared to those with leukaemias. This is unsurprising since the bone marrow is the primary site of involvement in the latter, and severe anemia, neutropenia and thrombocytopenia are present at diagnosis and expected, even in the absence of treatment.

Although the incidence of anaemia was higher in those with wasting using UAMA and MUAC, this association was lost in the adjusted model. Two studies from India found that children with undernutrition were more likely to be anaemic and receive red cell transfusions during the initial months of intensive chemotherapy [30, 31]. Many factors can account for anaemia in a child with cancer including bone marrow infiltration, cytokine release, chemotherapy-induced myelosuppression and bleeding [32]. Apart from cancer-related factors, low baseline haemoglobin levels, prior to development of cancer, contribute to increased transfusion requirements.

There was no significant association between nutritional status and the incidence of febrile neutropenia. Interestingly, prolonged neutropenia resulting in treatment delays was almost three times higher in those with average and low-average UAMA compared to those with severe wasting, remaining significant in multivariate analysis. The risk of neutropenia was also significantly lower among those whose MUAC indicated undernutrition. In contrast, Triarico et al in Italy found that in children ages 3–18 years, the rate of febrile neutropenia requiring at least three hospitalizations was significantly increased by underweight and rapid weight loss in the first six months after diagnosis [33]. In Nicaragua, Pribnow et al, in a retrospective review, also found that severe infection was more common in children, ages 6 months to 18 years, with undernutrition [9]. Additionally, Draper et al. in South Africa found that wasting in patients with Wilms tumour was significantly associated with treatment induced neutropenia [34]. Unlike the current study, their follow-up was for the entire duration of treatment allowing observation and evaluation of more febrile neutropenia events. The variation in our findings compared to other studies could be due to the practice where malnourished patients at the POU, KBTH are empirically given lower doses of chemotherapy or have longer durations between chemotherapy cycles to reduce the risk of toxicity. Those with normal nutritional status, therefore, receive relatively higher drug doses resulting in more profound neutropenia.

Children with low UAMA were more likely to develop oral mucositis while no episodes of mucositis in those with average UAMA. There is paucity of data in LMIC evaluating the risk of oral mucositis in children receiving chemotherapy. Oral mucositis results in pain, poor alimentation, prolonged hospital stay, increased cost of care, and treatment delays [35]. Malnutrition is a risk factor for mucositis which in turn can further worsen nutritional status due to reduced oral intake and poor nutrient absorption from damaged gastrointestinal surfaces [35]. Additionally, about 40% of children with mucositis in this study, concurrently developed febrile neutropenia. Disruption in mucosal barriers of the gastrointestinal tract allows pathogens to translocate into the blood resulting in an increased risk of septicaemia [36, 37].

Although proportionately more deaths occurred in study participants with wasting using arm anthropometry compared to those without wasting, this was not statistically significant. Studies in Malawi and India found that more deaths occurred in children with cancer who were wasted compared to those who were well-nourished although these were also not significant in multivariate analysis [8, 31, 38]. Other studies have shown results that are discrepant with ours [9, 39, 40]. The disparity could be due to longer follow-up periods, use of more intensive chemotherapy protocols, and retrospective study design with larger numbers of participants thus increasing the power to detect any association between acute undernutrition and mortality.

This study had several limitations, one of which is the short duration of follow up. It is also possible that a larger population size would have provided more power to detect associations. Additionally, different dosing regimens, patient groups and cancer types affect the ability to truly compare outcomes with other published studies.

## Conclusions

In conclusion, this study found that children with undernutrition identified by arm anthropometry at diagnosis of cancer had an increased risk of developing a complication within twelve weeks from the start of chemotherapy compared to those who were well-nourished. However, in multivariate analysis, only prolonged neutropenia remained significantly associated with UAMA and MUAC, ironically, occurring more frequently in those who were better nourished at cancer diagnosis. We attribute this, at least partly, to the local practice where chemotherapy regimens are dose-reduced for children with malnutrition in order to minimize toxicity. We recommend instituting nutritional assessment and rehabilitation programmes incorporating UAMA or MUAC at diagnosis of cancer for identification and monitoring of children with undernutrition. The International Society of Paediatric Oncology has published a framework for nutritional care in LMIC which supports the use of arm anthropometry for nutritional assessments in children with cancer. That document also provides guidelines for optimizing nutritional status through the course of treatment [41]. Further studies are needed to assess nutritional outcomes and evaluate complications beyond twelve weeks of chemotherapy initiation. Evidence is needed to support using lower dose treatment regimens for malnourished patients in settings with limited resources for supportive care.
